# An Exploratory Study of High-Concentration Trace Amine Effects and Adrenoceptor Expression Patterns in SH-SY5Y Cells and Neuroblastoma

**DOI:** 10.3390/ijms27115038

**Published:** 2026-06-02

**Authors:** Aleksandr V. Lopachev, Rogneda B. Kazanskaya, Raul R. Gainetdinov, Evgeny V. Kanov, Anastasia N. Vaganova

**Affiliations:** Institute of Translational Biomedicine, St. Petersburg State University, Universitetskaya Embankment. 7/9, 199034 St. Petersburg, Russia; a.lopachev@spbu.ru (A.V.L.);

**Keywords:** trace amines, neuroblastoma, adrenoceptors, monoamines, dopamine, GPCR

## Abstract

The antitumoral activity of monoamine receptor ligands appears to be important as a potential approach to cancer medication. In the present study, we evaluated the effects of trace amine compounds, including octopamine, tyramine, 3-methoxytyramine, and synephrine, on SH-SY5Y neuroblastoma cells. Previously, these compounds exhibited TAAR1-specific activity at nanomolar concentrations. However, in SH-SY5Y neuroblastoma cells, the effect of octopamine and 3-methoxytyramine was identified in concentrations of 1000 µM or above and is apparently non-specific. Neither public transcriptomic datasets nor qPCR analysis detected significant *TAAR1* expression in SH-SY5Y cells, suggesting that the observed effects may be mediated by adrenoceptors. Among these, *ADRA2C* was the most highly expressed in SH-SY5Y cells. Analysis of transcriptomic data from the GEO database revealed that adrenoceptors are widely expressed in neuroblastomas. The expression profiles of adrenoceptors in tumors are polymorphic and more complex than in SH-SY5Y cells. Thus, the effects of trace amines on other neuroblastoma cell lines and in vivo tumors warrant further investigation, and the involvement of adrenoceptors in this process may be speculated. Our findings suggest the non-specific activity of trace amines against tumor cells, with a paradoxical stimulatory effect in differentiated neuroblastoma cells, which highlights the need for caution in studies involving TAAR1-specific compounds.

## 1. Introduction

Neuroblastoma is a tumor that arises from primordial neural crest cells. This disease predominantly develops in infants and young children [1]. Neuroblastoma outcomes vary significantly among patients. In particular, in some pediatric patients it may fare well with little or no treatment, whereas older children with metastatic disease have a 50% event-free survival rate. In adults, the tumor is very rare, and the prognosis is substantially worse [2,3].

Both classical monoamines, such as dopamine or norepinephrine [4], and trace amines are produced by neuroblastoma cells, including m-octopamine, which is elevated in patients’ urine [5]. Thus, one of the hypotheses of neuroblastoma pathogenesis suggests that the tumor arises from the developing sympathoadrenal lineage and includes neural-like adrenergic and non-neuronal therapy-resistant mesenchymal cell populations [6]. Monoamine metabolites are widely used as biomarkers and prognostic indicators in neuroblastoma [7,8]. For example, higher levels of homovanillic acid, a dopamine metabolite, are associated with poor outcomes [9], and tumors with a dopaminergic phenotype show a similarly unfavorable prognosis [10].

While dopaminergic signaling is a recognized therapeutic target in cancer [11,12], the role of non-canonical monoamine signaling pathways in tumors, including neuroblastoma, remains unclear. Tyramine has been shown to be toxic to MAO-A-overexpressing neuroblastoma cells [13,14], likely due to oxidative mitochondrial DNA damage caused by its metabolism via monoamine oxidases A and B [15]. Whether other trace amines similarly influence neuroblastoma cell viability remains unknown.

The human neuroblastoma cell line SH-SY5Y is an established line deposited in the American Type Culture Collection (ATCC, Catalog No. CRL-2266). It was subcloned from the SK-N-SH cell line, which was isolated from the bone marrow biopsy of a 4-year-old female patient with metastatic neuroblastoma. Currently, these cells are applied for studies in neuroscience and for the modeling of biological processes in neurons. SH-SY5Y may be differentiated into a neuron-like phenotype in vitro. This model seems like a low-cost option compared to primary neurons in neurotoxicological studies, studies of neurodegenerative diseases, or neuroregeneration [16,17,18]. At the same time, SH-SY5Y cells are an in vitro model for neuroblastoma studies, including the measurement of anticancer compound activity [19,20].

SH-SY5Y cell line cultures consist of both adherent and floating cells and differentiate into two distinct phenotypes, i.e., neuroblast-like cells and epithelial-like cells [21]. These two phenotypes may correspond to the “N” and “S” types described in later studies in SH-SY5Y by Encinas et al. [22]. Cells with neuroblast-like morphology are positive for tyrosine hydroxylase (TH) and dopamine-β-hydroxylase, characteristic of catecholaminergic neurons, whereas the epithelial-like counterpart cells lacked these enzymatic activities [21]. SH-SY5Y cells can be differentiated to a more mature neuron-like phenotype that is characterized by neuronal markers. There are several methods to differentiate SH-SY5Y cells, and they are mentioned below. Retinoic acid (RA) is the most commonly used means for differentiation and will be addressed in detail [16].

Trace amines (TAs) represent a class of endogenous amine compounds found in mammalian neural tissues at trace concentrations (1–100 ng per gram), significantly lower than those of classical monoamine neurotransmitters such as catecholamines and serotonin. These compounds are detectable in synaptosomal fractions following tissue homogenization [23]. β-phenylethylamine, tyramine, tryptamine, octopamine, synephrine, and several other compounds are classified as trace amines [24,25]. Trace amine-associated receptors (TAARs) are the G-protein-associated receptors that recognize trace amines. TAAR1 is the most studied receptor in this family, which modulates dopaminergic neurotransmission [26] and is currently recognized as a promising target for the development of next-generation antipsychotic drugs [27,28,29]. However, TAAR1 ligands, like octopamine, could bind other receptors like α1- or α2-adrenoceptor subtypes, and the physiological importance of such interactions is not excluded [30,31,32].

The role of TAs and TAARs in cancer progression is an active area of research [33,34,35]. Antitumor effects have been reported for several TAAR1 ligands, including 3-iodothyronamine (T1AM) [36] and tryptamine [33]. Meanwhile, trace amines, like tyramine, may be carcinogenic by increasing DNA damage, stimulating cell proliferation, and inflammation [37]. In this study, we aimed to establish the sensitivity of SH-SY5Y neuroblastoma cells to the natural trace amines tyramine (Tyr), synephrine (Syn), octopamine (Oct), and dopamine metabolite 3-methoxytyramine (3-MT) in supra-physiological concentrations and provide a comparative expression profile of their receptors, i.e., TAARs and adrenoceptors, in SH-SY5Y cells and neuroblastoma clinical samples harvested from patients.

## 2. Results

### 2.1. Influence of Compounds Under Study on the Viability of Differentiated and Undifferentiated SH-SY5Y Cultures

**Figure 1:**
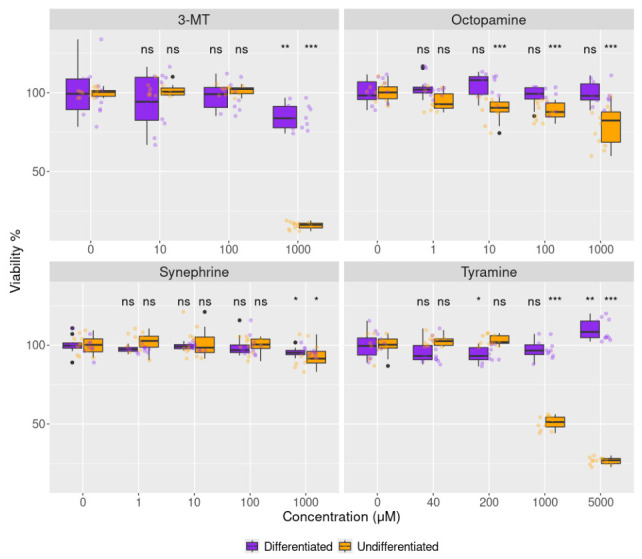
Box plots of differentiated and not differentiated SH-SY5Y neuroblastoma culture viability after 48 h incubation with different concentrations of 3-methoxytyramine, octopamine, synephrine, and tyramine. Boxes extend from the 25th to the 75th of each group’s distribution of values. Horizontal lines denote median values. Vertical lines represent the 1.5 interquartile range of the 25th to the 75th percentile of each group. Dots represent individual values, outliers marked as black dots. Data is presented as % of solvent control, *—*p* < 0.05, **—*p* < 0.01, ***—*p* < 0.001 (Tukey post hoc test against zero compound concentration). Cell viability is presented as % of solvent control.

We estimated the effect of natural TAAR1 ligands Tyr, Oct, Syn, and 3-MT on the undifferentiated and differentiated SH-SY5Y cells’ viability. As presented in Figure 1, naturally occurring trace amines Tyr, Oct, Syn, and 3-MT differently affect the viability of the SH-SY5Y culture. 48 h incubation with 1 mM 3-MT caused an 84% decrease (*p* < 0.001) in undifferentiated culture viability and only a 15.2% (*p* < 0.001) decrease in differentiated culture viability (Figure 1).

Oct causes a decrease in undifferentiated culture viability in a dose-dependent manner; in the lowest concentration among the listed compounds above, 10 µM, by 10.6%, *p* < 0.01. Meanwhile, 100 µM and 1 mM Oct caused only a 12% (*p* < 0.01) and 21% (*p* < 0.001) decrease in undifferentiated culture viability, respectively. Oct up to 1 mM has not caused any decrease in differentiated culture viability (Figure 1).

Additionally, 1 mM Syn caused only a slight, 6.7% (*p* < 0.05) decrease in undifferentiated culture viability and did not affect differentiated ones (Figure 1).

A 48 h incubation with 1 mM and 5 mM Tyr caused a 49.2% (*p* < 0.001) and 73.4% (*p* < 0.001) decrease in undifferentiated culture viability. In contrast, only 5 mM Tyr caused a slight but significant, 9% (*p* < 0.001), increase in differentiated culture viability (Figure 1).

Thus, we can conclude that an undifferentiated SH-SY5Y neuroblastoma culture is more sensitive to naturally occurring trace amines than a differentiated one. However, only octopamine caused the decrease in undifferentiated culture viability in micromolar concentrations.

**Figure 2:**
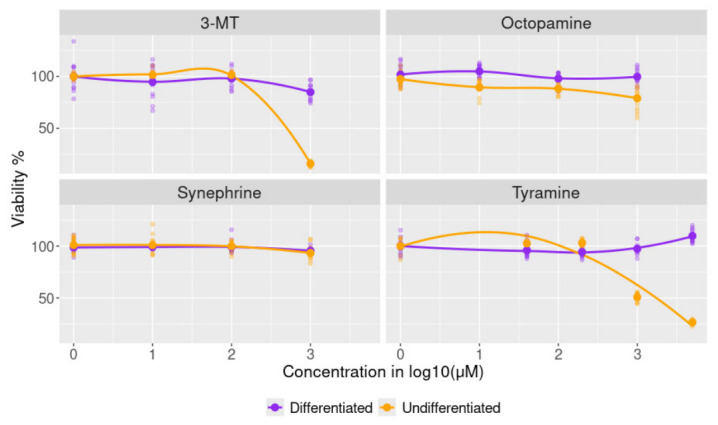
Dose–response curves of cytotoxic effect (if occurred) in differentiated and non-differentiated SH-SY5Y neuroblastoma cells after 48 h incubation with serial dilutions of 3-methoxytyramine hydrochloride, octopamine hydrochloride, synephrine, and tyramine hydrochloride. Cell viability is presented as % of solvent control. Dots represent individual values.

Dose–response curves show (Figure 2), however, in the experimental conditions, only Tyr exerted some cytotoxic effect upon undifferentiated cells (IC50 = 1433 µM). 3-MT (Figure 2) also lowered the viability of these cells, but its IC50, due to bad curve fit and a wide 95% CI, cannot be determined properly, as well as trace amine effects in differentiated cultures.

### 2.2. TAARs and Adrenoceptors Expression in Undifferentiated SH-SY5Y

Considering the low but identifiable trace amine effect against SH-SY5Y neuroblastoma cells, we considered it may be related to their nonspecific effect on adrenoceptors. So we estimated the repertoire of these GPCRs in the SH-SY5Y cell line by RT-PCR and in silico methods.

Following the inclusion criteria listed in paragraph 4.5, two GEO RNA-seq transcriptomic datasets listed in Table 1 were included in the comparative secondary analysis. Both datasets were generated by RNA sequencing using the Illumina HiSeq 4000 platform (San Diego, CA, USA).

**Figure 3:**
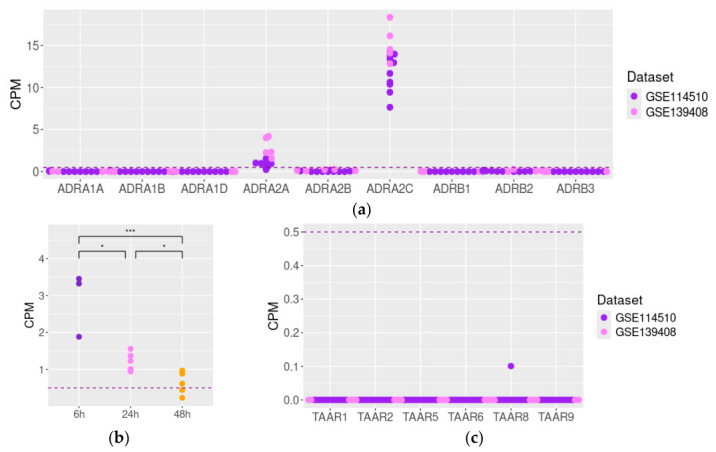
Adrenoceptors (**a**,**b**) and TAARs (**c**) expression in the intact undifferentiated SH-SY5Y cells in the datasets included in the analysis, and data were CPM-normalized, with a cut-off level of 0.5 CPM represented by the dotted line. Figure 3b demonstrates *ADRA2A* mRNA expression in SH-SY5Y cultures after different growth times (GSE114510). Dots represent individual values: *—*p* < 0.05, ***—*p* < 0.01. 6 h, 24 h, and 48 h—6, 24, and 48 h from the beginning of the experiment.

In both datasets included in the analysis, count per million (CPM) normalized expression levels demonstrated the congruent adrenoceptor expression pattern. We assessed α- and β-adrenoceptor expression in the selected datasets (Figure 3a). The expression of *ADRA2C* mRNA was identified in all studied samples in both datasets. Also, *ADRA2A* mRNA expression was revealed in SH-SY5Y samples represented in the GEO repository (Figure 3a), but its level was pronouncedly lower than the ADRA2C expression level (1.79 and 12.74 CPM, respectively).

The dataset GSE114510 consists of the data of RNA sequencing of SH-SY5Y cells harvested at different time points after the beginning of observation. The analysis of adrenoceptors’ mRNA expression in the intact cells in this dataset revealed the gradual decrease in *ADRA2A* mRNA expression throughout 48 h of observation (*p* < 0.05, Figure 3b).

No expression of TAAR-family receptors was identified in both datasets included in the analysis (Figure 3c).

### 2.3. α-Adrenoceptors Expression Does Not Change in SH-SY5Y After Differentiation

**Figure 4:**
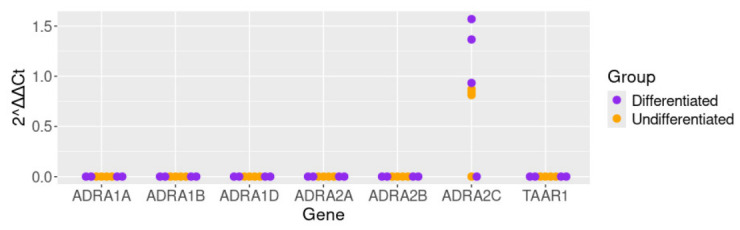
Reverse transcription–polymerase chain reaction (RT-qPCR) confirmed ADRA2C mRNA expression in the SH-SY5Y cells. *ADRA2C* expression levels were normalized against expression in undifferentiated cells.

We further assessed α-adrenoceptor genes’ expression in the SH-SY5Y cells using RT-qPCR (Figure 4). The RT-qPCR confirmed *ADRA2C* mRNA expression in both the differentiated and undifferentiated SH-SY5Y neuroblastoma cells. The expression levels were comparable in both states, with slight, insignificant (*p* > 0.05) upregulation in differentiated SH-SY5Y cells compared to the undifferentiated culture. *TAAR1 mRNA* expression was not detected in either undifferentiated or differentiated SH-SY5Y cells (n = 4 for each condition).

### 2.4. Adrenoceptors mRNA Repertoire in Neuroblastoma

To estimate the adrenoceptor expression in the neuroblastoma samples obtained at different disease stages, including disseminated tumor cells in patients with metastatic diseases, we analyzed three transcriptome RNA-generated datasets (Table 2).

**Figure 5:**
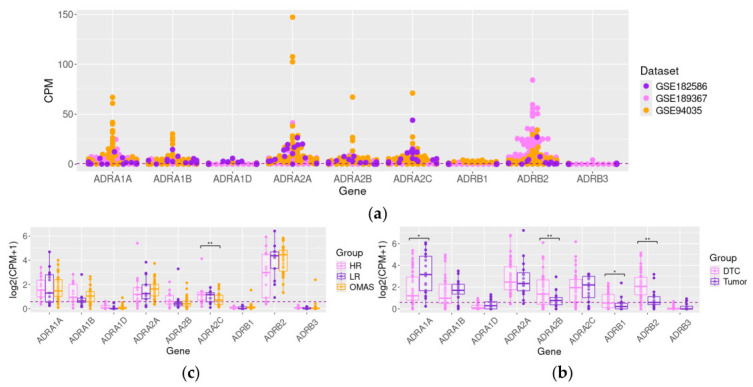
Adrenoceptors’ mRNA expression in neuroblastoma samples. (**a**) count per million (CPM)-normalized expression levels of adrenoceptors’ mRNA in patients’ samples; (**b**) expression levels in primary Stage IV tumors and disseminated tumor cells (DTC) isolated from Stage IV neuroblastoma patients’ bone marrow (GSE94035); (**c**) comparative expression levels in tumors from low-risk (LR) or high-risk (HR) patients without opsoclonus-myoclonus ataxia syndrome (OMAS) and tumors from OMAS patients (GSE189367). *—*p* < 0.05, **—*p* < 0.01.

The extracted data demonstrated variable adrenoceptor expression in tumor samples (Figure 5a). *ADRA1A*, *ADRA1B*, *ADRA2A*, *ADRA2B*, *ADRA2C*, and *ADRB2* mRNA demonstrated more pronounced expression in datasets included in the analysis. The mRNA of these genes was identified in 83.1%, 80.1%, 91.2%, 58.1%, 78.7%, and 89%. At the same time, *ADRA1D*, *ADRB1*, and *ADRB3* mRNA were identified in 18.4%, 20.6%, and 5.1% of samples, respectively. Thus, the majority of tumors demonstrate a more complex adrenoceptor repertoire compared to SH-SY5Y neuroblastoma cells, i.e., more than two adrenoceptors.

Additionally, we revealed the association between *ADRA2C* mRNA expression decrease and opsoclonus-myoclonus ataxia syndrome development (*p* < 0.01), as well as decreased *ADRA1A* and increased *ADRA2B*, ADRB1, and *ADRB2* mRNA expression in the disseminated neuroblastoma cells compared to the primary tumor, which confirms possible involvement of adrenergic signaling in the neural crest cells’ malignization.

## 3. Discussion

In the present study, the activity of four natural TAAR1 ligands was tested against neuroblastoma SH-SY5Y cells in vitro. In this study, we utilized SH-SY5Y cells in two states: undifferentiated proliferating cells and cells treated with RA. The RA treatment was employed to achieve a differentiated-like model exhibiting key neural features, such as the development of branched neurites. Critically, this latter model represents a less aggressive cancer phenotype compared to naive SH-SY5Y cells, allowing us to evaluate the targets across different stages of malignancy. All four ligands demonstrated slight suppressive activity against not-differentiated neuroblastoma cells; however, this effect became pronounced only when the ligands’ concentrations were several times higher than necessary to activate the TAAR1 receptor. The concentrations of trace amines employed in this study (micromolar to millimolar range) allow exploration of the metabolic and survival limits of SH-SY5Y cells. While TAAR1-mediated signaling is typically characterized in the nanomolar range, higher concentrations are often required to engage a wider spectrum of adrenoceptors and metabolic pathways in vitro, particularly in cancer models where receptor density and metabolic flux may be altered. In particular, the decreased effect on SH-SY5Y cell viability was demonstrated for concentrations of 1000 μM or above of TYR, SYN, and byproduct of dopamine metabolism, 3-MT, despite the EC50 values for TAAR1-dependent stimulation of cAMP formation being 1 μM Tyr [41], 1.8 μM 3-MT, or 23 μM Syn [40]. Oct demonstrated weak activity against SH-SY5Y in concentrations near the identified IC50 of 7.6 ± 0.80 μM [40]. However, only Tyr exerted a cytotoxic effect against undifferentiated cells, which was sufficient to estimate its IC50, which was 1433 µM.

Furthermore, we revealed that the effect of all ligands except Tyr was less pronounced in differentiated SH-SY5Y cells compared to undifferentiated cultures. Differentiation of neuroblastoma cells in vitro is associated with decreased cell sensitivity to cytostatics, including doxorubicin, melphalan, and 5-fluorouracil [42]. This effect, at least in SH-SY5Y cells, is mediated by the reduction in the apoptotic response to antineoplastic drugs [43]. Differentiated SH-SY5Y cells are also more resistant to heavy metals [44] or the natural compound streptozotocin, which has broad-spectrum antibiotic activity and antineoplastic properties [45]. Interestingly, we identified a paradoxical effect of Tyr in the differentiated neuroblastoma cells SH-SY5Y. Despite its inhibitory effect in undifferentiated cultures, in differentiated cells it stimulated ATP production.

Thus, the effect of TAAR1 ligands was very weak and became detectable only when ligand concentrations were significantly higher than EC50, previously described for this receptor [40,41]. We also did not detect *TAAR1* mRNA expression in undifferentiated or differentiated SH-SY5Y cells, either in public transcriptomic data or in our RT-PCR. These results indicate that the effect of TAAR1 ligands in neuroblastoma depends on some non-specific interactions. Previously, non-specific interactions between adrenoceptors and trace amines were identified [30,31,32]. Syn was suggested to act as an antagonist of ADRA2C [46]. However, other studies refer to it as a weak α-adrenoceptor agonist [47]. Tyr’s adrenomimetic properties have also been demonstrated previously [48,49]. Also, the suggestion that α-adrenoceptors are candidates for mediating trace amine effects is supported by their status as vertebrate orthologs of the invertebrate tyramine receptor, which share a common evolutionary origin and functional similarity with the ancestral tyramine-signaling system [50,51]. So it may be speculated that the identified effect may be mediated by the adrenoceptors.

In accordance with previous data [52,53], we confirmed that the α2-adrenoceptor ADRA2C is expressed in both undifferentiated and differentiated SH-SY5Y cells [52]. Adrenergic signaling may promote neoplastic transformation and participate in conditioning the local microenvironment for colonization by cancer cells. The adrenergic system also modulates the immune system in the tumor microenvironment [54,55]. ADRA2C has been identified in tumor samples from different tissue origins. Moreover, it was found that ADRA2C expression may be associated with the prognosis in some cancers like adrenocortical carcinoma, glioblastoma, glioma, or uveal melanoma [56].

It has been shown that the ADRA2A agonist clonidine promotes cell proliferation in triple-negative breast cancer cells, and the ADRA2A antagonist rauwolscine diminishes tumor growth in vitro and in vivo [57]. Also, dexmedetomidine, which is used for intraoperative sedation, is a highly selective ADRA2A agonist that reinforces the malignant behavior of lung, colorectal [58], or breast cancer cells [59]. On the other hand, clonidine suppresses pancreatic ductal carcinoma cell invasion [60]. So, the α2-adrenoceptor’s role in tumors seems to be context-dependent.

Previously, it was revealed that several compounds act on the α2-adrenoceptor, which acts through adrenoceptors, and demonstrate an antiproliferative effect. Adrenoceptors mediate effects against neuroblastoma, both in vitro and in xenograft models in vivo [61,62]. In a neuroblastoma patient group study, ADRB2 is a protective biomarker that is associated with a better survival rate [63]. Meanwhile, some effects on the prognosis and survival rate may be related to adrenoceptors’ expression on the stromal cells, at least on the immune cells, rather than on tumor tissue [64]. In this study, we utilized the SH-SY5Y cell line, which allowed for a controlled and reproducible tool. Acknowledging that a single cell line cannot fully capture the complex phenotypic heterogeneity inherent in neuroblastoma, we estimated adrenoceptors’ expression in public transcriptomic data derived from pathological samples. The study of three transcriptomic datasets, which altogether include 96 tumor samples and 40 disseminated tumor cells, demonstrates that expression levels of adrenergic receptors vary and may depend on tumor characteristics like the degree of malignancy or other properties, which remain unidentified. Such variability also may partially explain the discrepancies in ADRA2A expression levels in two datasets included in the analysis.

This study has several limitations that should be considered when interpreting the results. mRNA levels serve only as an indirect indicator of receptor expression and functional activity. Therefore, this study provides only indirect evidence of adrenoceptor involvement in the response of SH-SY5Y cells to TAAR1 ligands. Moreover, the cytotoxic potency of the trace amine compounds tested is very weak by itself, and, in any case, not sufficient for practical needs. Due to the limited availability of suitable datasets in the GEO repository, our ability to analyze associations between adrenoceptor expression and tumor characteristics (such as grade or TNM stage) was also restricted. Consequently, it is not possible to draw conclusions about the significance of adrenoceptor expression in the development and progression of neuroblastoma based on the current data. While the MTT assay is a valuable tool for viability screening, its reliance on mitochondrial dehydrogenase activity can lead to skewed results at high ligand concentrations. Such concentrations may induce direct chemical interference, metabolic shifts, or excessive ROS production, potentially leading to the non-enzymatic reduction in MTT or the inhibition of mitochondrial dehydrogenases [65]. While our findings suggest their involvement, we did not directly measure downstream signaling events, such as cAMP accumulation or G-protein activation. Future studies employing selective agonists, antagonists, or signaling assays are required to definitively confirm the receptor-mediated nature of these effects.

Furthermore, the dose dependency observed in our experiments warrants a cautious interpretation. At the higher concentration range of tyramine, it is possible that the observed effects are not exclusively driven by adrenoreceptor activation. In particular, the paradoxical 9% increase in ATP levels observed in differentiated cells presents a possible manifestation of this mechanism. As a biogenic amine, tyramine is a substrate for monoamine oxidases (MAO), and its rapid metabolism can lead to the accumulation of hydrogen peroxide and other reactive oxygen species, potentially inducing oxidative stress or metabolic toxicity. Moreover, both undifferentiated and differentiated SH-SY5Y cells are producers of MAOA [66]. We hypothesize that identified increases in mitochondrial dehydrogenase activity may be driven by ROS generated by MAOA, acting at low concentrations as intracellular signaling molecules rather than destructive agents. This concept aligns with the principle of mitohormesis, where oxidative stress triggers adaptive, compensatory responses that enhance cellular bioenergetics and survival pathways [67]. In differentiated neuroblastoma cells, such low-level MAO-derived ROS signaling could stimulate mitochondrial biogenesis or optimize the efficiency of the electron transport chain. The potential contribution of these receptor-independent pathways should be addressed in future research using MAO inhibitors or antioxidants. Meanwhile, an increase in metabolic activity (MTT signal) in differentiated cells treated with 5 mM tyramine suggests a more complex biological response, like adrenomimetic metabolic activation or stimulation of pro-survival pathways, such as those mediated by Bcl-2 induced by MAO-produced ROS [68].

Overall, despite all the limitations described above, we demonstrated an inhibitory effect of trace amine against neuroblastoma cells, which seems to be associated with some unspecific interactions. While our data suggest a putative link between the observed effects and the ADRA2C receptor, we acknowledge that these findings are preliminary and correlative. However, the analysis of adrenoceptor expression in neuroblastoma revealed that their pattern in these tumors is heterogeneous and more complex than in SH-SY5Y cells cultured in vitro. So the adrenoceptor-related effects in other cell lines of primary cells and tumors may be different from those described in the present study. Further functional validation, including receptor blockade or genetic silencing, is essential to definitively establish the specific contribution of ADRA2C versus non-specific cytotoxic effects.

## 4. Materials and Methods

### 4.1. Cells

The SH-SY5Y (ATCC, Manassas, VA, USA) human neuroblastoma cell line was maintained in MEM with Earle’s salts/F12 medium (PanEco, Moscow, Russia) supplemented with 10% fetal calf serum, 2 mM glutamine, and 100 U/mL penicillin-streptomycin (complete culture medium—CCM) in a humidified incubator at 37 °C and 5% CO_2_ as 80% of confluent monolayer in 100 mm diameter tissue culture dishes. Culture was incubated in MEM with Earle’s salts/F12 medium supplemented with 1% fetal calf serum and 10 μM RA for 7 days to induce differentiation.

### 4.2. Compounds

Tyramine hydrochloride (Tyr) (Sigma-Aldrich, St. Louis, MO, USA) was diluted at final concentrations of 40, 200, 1000, and 5000 µM; 3-methoxytyramine hydrochloride (3-MT) (Sigma-Aldrich, St. Louis, MO, USA) at 10, 100, and 1000 µM; octopamine hydrochloride (Oct) (Sigma-Aldrich, St. Louis, MO, USA) at 1, 10, 100, and 1000 µM; and synephrine (Syn) (Sigma-Aldrich, St. Louis, MO, USA) at 1, 10, 100, and 1000 µM. All dilutions were made in CCM with 0.5% (*w*/*v*) Tween-20, which resulted in a <0.01% final concentration of solubilizer in the incubation medium. In preliminary experiments we found that Tween-20 in concentrations of 0.01% and less did not affect the viability of SH-SY5Y cells (Figure S1 and Table S1 in Supplementary Materials).

### 4.3. MTT Assay

Undifferentiated and differentiated cells were seeded at 2.5 × 10^4^/well in 96-well flat-bottomed tissue culture-treated microtiter plates and cultured for 24 h. After that, serial dilutions of compounds under study (see above) were added in corresponding wells, and plates were then incubated at 37 °C and 5% CO_2_ for an additional 48 h. Equal volumes of CCM with 0.5% (*w*/*v*) Tween-20 were added to the solvent control wells. At the end of the incubation period, the culture medium was discarded, and 50 µL of MTT (Sigma-Aldrich, St. Louis, MO, USA) solution (0.5 mg/mL final concentration) was added to each well, and the plates were incubated for 2 h in a humidified incubator at 37 °C and 5% CO_2_. After the incubation, water-insoluble formazan crystals were dissolved by the addition of 50 µL ≥99% dimethyl sulfoxide (Sigma-Aldrich, St. Louis, MO, USA), and absorbance was measured at 540 nm using a Synergy H1 plate reader (Bio-Tek Instruments, Charlotte, VT, USA). Cell viability in experimental wells was calculated as a percentage of optical density in solvent control wells (taken as 100% viability). Twelve independent passages (biological replicates) were applied in each experiment.

### 4.4. RNA Isolation, Reverse Transcription, and Quantitative Polymerase Chain Reaction (qPCR)

Cells were plated according to the above protocol into 6-well plates at a density of 2 million per well in the medium. 2 mL of 0.05% trypsin-EDTA with Hank’s salts (PanEco, Moscow, Russia) was added to the cells, and the cells were incubated for 5 min at 37 °C and then suspended by pipetting in 5 mL of the medium. The resulting suspension was centrifuged for 2 min at 400 g. The resulting pellet was resuspended in Hank’s balanced salt solution without Ca^2+^ and Mg^2+^ (PanEco, Moscow, Russia) and centrifuged for 2 min at 400 g. RNA was isolated from the resulting pellet. Each condition (undifferentiated and differentiated) included four independent passages as biological replicates.

Total RNA was extracted by the RNA Solo kit (Evrogen, Moscow, Russia) following the manufacturer’s protocol. RNA concentration and purity were assessed using a NanoDrop 2000 Spectrophotometer (Thermo Fisher Scientific, Waltham, MA, USA) with 2 µL of RNA solution. For reverse transcription, 0.3 µg of RNA was used. Complementary DNA (cDNA) synthesis was performed using the MMLV RT kit (Thermo Fisher Scientific, Waltham, MA, USA) according to the manufacturer’s instructions.

Gene-specific primers utilized in this study are provided in Table 3. Each cDNA sample underwent at least two independent qPCR runs (technical replicates) using the QuantStudio™ 5 Real-Time PCR System (Applied Biosystems, Waltham, MA, USA). Transcript levels were quantified with qPCRmix-HS (Evrogen, Moscow, Russia).

The qPCR protocol consisted of 40 thermal cycles, each including denaturation at 95 °C for 10 s, annealing at 60 °C for 15 s, and elongation at 72 °C for 30 s, followed by fluorescence detection. To verify amplicon specificity, a melting curve analysis was performed for each amplification product using the default protocol of the QuantStudio™ 5 Real-Time PCR System (Applied Biosystems, Waltham, MA, USA). A maximal Ct value cut-off of 33 was used to determine positivity for all RT-PCR reactions.

Relative gene expression levels were determined using the 2^−ΔΔCt^ method. The analysis proceeded as follows: Cycle threshold (Ct) values were first recorded for each sample (i.e., mean value between two technical replicates). ΔCt values were then calculated by normalizing the Ct of the target gene to the Ct of the housekeeping gene (*GAPDH*). Next, the mean Ct value for undifferentiated cells was subtracted from each ΔCt to yield ΔΔCt. Finally, relative expression levels were derived by calculating 2^−ΔΔCt^.

### 4.5. Public Data Collection and Inclusion Criteria

The expression data for SH-SY5Y cells were obtained from transcriptome datasets available in the Gene Expression Omnibus (GEO) database [69]. Datasets generated by RNA sequencing were selected based on the following criteria: (1) availability of expression RNA-sequencing data in raw counts; (2) inclusion of at least five biological replicates per study group; and (3) due to the low transcription levels of GPCRs, only samples with a minimum of 25 million reads in SRA files were considered. Additionally, we include in the analysis three GEO datasets representing data obtained by RNA-sequencing studies of tumor samples (GSE94035, GSE182586, and GSE189367).

### 4.6. Public Data Processing and Analysis

Raw count data were retrieved from the NCBI GEO repository and normalized to counts per million (CPM) using the edgeR package (version 4.6.2) [70], with CPM values exceeding a threshold of 0.5 considered positive expression (taking into account the inclusion criteria, the expression level of 0.5 CPM was above 10 raw counts). Since GPCRs are notoriously low-expressed, we select the low threshold to escape losing biologically meaningful data [71,72]. Visualization of expression data was performed using the ggplot2 package (version 3.5.2) [73].

Differential gene expression analysis was conducted using a quasi-likelihood F-test in edgeR (version 4.6) [70]. To account for multiple testing, *p*-values were adjusted using the Benjamini–Hochberg procedure. Genes with an adjusted *p*-value < 0.05 were classified as differentially expressed.

### 4.7. Statistics

MTT assay results were presented as a percentage of cell viability in solvent control wells (taken as 100%). For half-maximal inhibitory concentration (IC50) evaluation, five-point dose–response curves were analyzed in GraphPad Prism 8.0 (GraphPad Software, Boston, MA, USA) by means of non-linear curve fitting. Sample distribution analysis was performed using the Shapiro–Wilk normality test. Statistical analysis was performed by two-way ANOVA with Tukey post hoc tests.

Each gene expression level assay was conducted in duplicate, and mean values were used for subsequent statistical analysis. Data were assessed for normal distribution using the Shapiro–Wilk test. Differences in normalized expression levels were evaluated with either a Student’s *t*-test (for normally distributed data, where *p* > 0.05 across all groups) or a Mann–Whitney U test in R (v.4.5.3).

The results were visualized with the ggplot2 R package (v. 3.5.2.) [73].

## 5. Conclusions

In conclusion, the findings presented in this study confirm the very slight inhibitory effect of trace amines on neuroblastoma cell viability. We propose that these observed effects may be partially mediated through the ADRA2C adrenergic receptor, highlighting a potential pharmacological target for further investigation in the context of neuro-oncology. However, it is imperative to acknowledge that this suggestion remains preliminary, suggestive, and based on the mechanistic link; thus, we emphasize that this hypothesis is based on indirect evidence. Also, the transition from in vitro mechanistic observations to broader clinical implications is complicated by the inherent biological diversity of the disease. Our analysis of adrenoceptor expression patterns in neuroblastoma clinical samples reveals a high degree of heterogeneity, suggesting a regulatory landscape that is significantly more intricate than that observed in the standardized SH-SY5Y cell line. Consequently, while the SH-SY5Y model serves as a valuable tool for initial screening and mechanistic studies, the adrenoceptor-related effects identified here may manifest differently in other cell lines, primary patient-derived cultures, or various subtypes of primary tumors. Future research should prioritize functional validation using a broader range of experimental models and high-resolution expression profiling. Such studies are essential to fully elucidate the therapeutic potential of trace amine-mediated pathways and to account for the complex molecular architecture of neuroblastoma in a clinical setting.

## Figures and Tables

**Table 1:** RNAseq datasets included in the analysis of the SH-SY5Y transcriptome.

Samples Included in the Analysis	Samples Characteristics	Title	Dataset ID
Untreated control samples were included in the analysis (*n* = 13)	SH-SY5Y cells were treated with GM6 or water (control). Cells were sampled at 6 h, 24 h, and 48 h [38]	GM604 regulates developmental neurogenesis pathways and the expression of genes associated with amyotrophic lateral sclerosis	GSE114510
Untreated control samples were included in the analysis (*n* = 6)	SH-SY5Y were cultured in conditions of intermittent exposure to ethanol for four weeks or grew without ethanol for the same time [39]	Effects of chronic intermittent ethanol exposure and withdrawal on neuroblastoma cell transcriptome	GSE139408

**Table 2:** RNAseq datasets included in the analysis of the neuroblastoma transcriptome.

	*n*	Platform	Title	Dataset ID
Tumor, Stage I, II	10	Illumina NovaSeq 6000	Tumor Microenvironment Profiling Identifies Prognostic Signatures and Suggests Immunotherapeutic Benefits in Neuroblastoma	GSE182586
Tumor, Stage III, IV	6			
Tumor, Stage III, IV (high-risk group following [40])	13	Illumina NovaSeq 6000	Polyclonal lymphoid expansion drives paraneoplastic autoimmunity in neuroblastoma [RNA-Seq]	GSE189367
Tumor, Stage I, II (low-risk group following [40])	13			
Tumor, Stage I-IV from patients who developed opsoclonus-myoclonus ataxia syndrome (refer for details [40])	38			
Primary tumor, Stage IV	16	Illumina HiSeq 2000	Neuroblastoma cells undergo transcriptomic alterations during dissemination into the bone marrow and subsequent tumor progression	GSE94035
Disseminated tumor cells form bone marrow, Stage IV	40			

**Table 3:** Primers and probes were used for quantitative RT-PCR.

Gene	Reverse Primer	Forward Primer
*ADRA1A*	GTG GAA TAT GTG CTG AGA CCC A	AGG ACA AGG ATT TGG TGC CTC G
*ADRA1B*	GCA TGT TGC TTT TGA AGC CC	ACA AAC ACC CTC CTT CTG GC
*ADRA1D*	GAG GAA GGC GCG CTT GAA CTC	CAT CGT CGT GGG TGT CTT CGT G
*ADRA2A*	TGG TAG ATG CGC ACG TAG AC	CTC CAT CGA GAA GAA GGG CG
*ADRA2B*	ACA AAC ACC CTC CTT CTG GC	AAC GGA CAC TCG AAG TCC AC
*ADRA2C*	TTC AGG TTG TAC TCG ACG GC	CTG GTC ATG CCC TTC TCG TT
*TAAR1*	ACA GTG CTC AGC AGA TCT CAC CA	TGA CCA CAC TCG TTG GCA ACT TG
*GAPDH*	AGG GGC CAT CCA CAG TCT TCT G	CAC CAC CAA CTG CTT AGC ACC C

## Data Availability

The original contributions presented in this study are included in the article/Supplementary Materials. Further inquiries can be directed to the corresponding author.

## Supplementary Materials

The following supporting information can be downloaded at: https://www.mdpi.com/article/10.3390/ijms27115038/s1.

## Abbreviations

The following abbreviations are used in this manuscript:

3-MT	3-methoxytyramine
CPM	Count per million
GEO	Gene Expression Omnibus
IC50	Inhibitory concentration 50
Oct	Octopamine
RT-PCR	Reverse transcription-polimerase chain rection
Syn	Synephrine
Tyr	Tyramine
