# Supplementary material for: An Exploratory Study of High-Concentration Trace Amine Effects and Adrenoceptor Expression Patterns in SH-SY5Y Cells and Neuroblastoma
Source: Int J Mol Sci. 2026 Jun 2;27(11):5038. doi: 10.3390/ijms27115038 (PMC13256704; doi:10.3390/ijms27115038)
Supplement: Supplementary file 1 [file ijms-27-05038-s001.zip › ijms-4319249-supplementary.pdf]

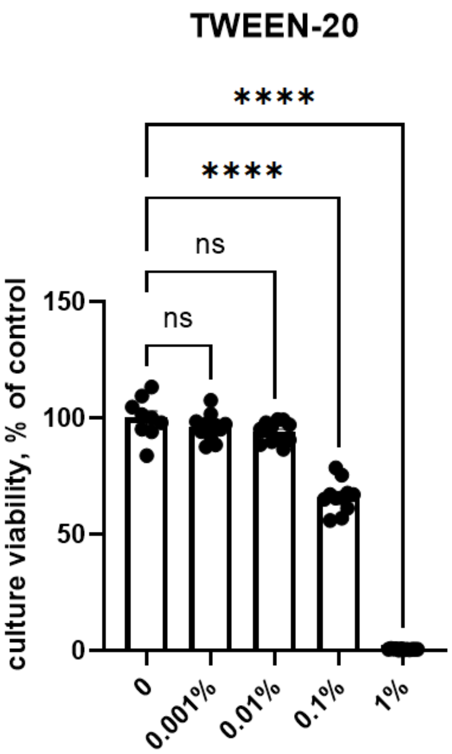

Figure S1. Viability of SH-SY5Y incubated with different % of Tween-20 according to MTT-test. N=9-11, **** - p<0.001

Table S1. Viability of SH-SY5Y incubated with different % of TWEEN-20 according to MTT-test.

**Tween-20**	**0**	**0.001%**	**0.01%**	**0.1%**	**1%**
Number of values	9	10	10	11	11
Mean	100	95.63	94.47	66.02	0.5984
Std. Deviation	8.76	3.884	4.149	6.795	0.1989
Std. Error of Mean	2.92	1.228	1.312	2.049	0.05996
**Shapiro-Wilk test**					
W	0.9816	0.9483	0.8852	0.9291	0.9003
P value	0.9721	0.6489	0.1497	0.4019	0.1863
Passed normality test	Yes	Yes	Yes	Yes	Yes
**Dunnett's test**	**Adjusted P Value**				
0 vs. 0.001%	0.2457				
0 vs. 0.01%	0.1002				
0 vs. 0.1%	<0.0001				
0 vs. 1%	<0.0001				
